# Clinical utility of serum cystatin C for prediction of multi-vessel disease by coronary angiography in type 2 diabetes mellitus patients with normal renal function

**DOI:** 10.1186/s12872-020-01475-4

**Published:** 2020-04-19

**Authors:** Shaoyi Wang, Qiaohui Liu, Fangfang Guo, Xiaocong Zhou, Jie Shi, Qing Xie

**Affiliations:** 1 Department of Orthopedics, Qilu Hospital of Shandong University, Jinan, China; 2 School of Medicine, Shandong University, Jinan, China; 3 Pharmacy Department, Qilu Hospital of Shandong University, No.107, Wen Hua Xi Road, Jinan, Shandong 250012 People’s Republic of China; 4 Shandong Provincial Qianfoshan Hospital, Jinan, China

**Keywords:** Cystatin C, Gensini score, Diabetes mellitus, Multivessel disease

## Abstract

**Background:**

The aim of this study was to evaluate whether serum cystatin C could serve as a predictor of multivessel coronary artery disease identified by coronary angiography in type 2 diabetes patients with normal renal function and to suggest the cutoff point of serum cystatin C for predicting multivessel disease.

**Methods:**

Serum cystatin C concentrations were measured by using particle-enhanced immunonephelometric assays before coronary angiography in 135 consecutive type 2 diabetes patients and 179 nondiabetic patients with normal renal function. Routine anthropometric and serologic data were collected. The severity of multivessel disease was assessed with the Gensini score after coronary angiography. The associations of serum cystatin C with the Gensini score were investigated, and the independent risk factors associated with multivessel disease were assessed.

**Results:**

Serum cystatin C and the Gensini score were significantly elevated in diabetes patients. Cystatin C had a positive correlation with Gensini score. A multiple logistic regression analysis demonstrated that cystatin C was independently associated with the presence of multivessel disease (the OR score is 2.21, *P* = 0.003). Based on the ROC curve, a cystatin C level of 0.865 mg/L showed 67.7% sensitivity and 76.3% specificity with an AUC of 0.748 in diabetes patients for detecting multivessel disease.

**Conclusion:**

Serum cystatin C is significantly correlated with the presence of multivessel disease, suggesting that cystatin C might be utilized as a screening tool for predicting multivessel disease in type 2 diabetes mellitus patients with normal renal function.

## Introduction

Type 2 diabetes mellitus is common and an important cause of morbidity and mortality. Type 2 diabetes mellitus is associated with accelerated atherosclerotic processes affecting arteries that supply major organs in the body. Therefore, patients with diabetes are at high risk for cardiovascular diseases, stroke and renal dysfunction [1, 2]. A series of studies have demonstrated that multiple factors, including hyperglycaemia, fluctuating blood glucose levels, central obesity, and hyperlipidaemia, contribute to the increased risk of CAD (cardiovascular diseases) [3, 4]. The link between hyperglycaemia and cardiovascular disease is well documented, and there is ample epidemiological evidence that hyperglycaemia is an independent predictor of cardiovascular mortality in patients with DM (diabetes mellitus) [5].

Multivessel disease is the most fatal and frequently observed coronary artery disease in type 2 diabetes patients. Until now, no simple, noninvasive screening tool has been available. The close correlation between multivessel disease and fluctuating blood glucose levels in type 2 diabetes patients has been shown in clinical and experimental studies [6]. However, the detailed mechanisms underlying the link between multivessel disease identified by coronary angiography and type 2 diabetes mellitus remain to be elucidated. Fluctuating blood glucose levels cannot fully explain the close association.

Cystatin C, which is less influenced by sex, age and race, has been considered a novel sensitive marker for detecting renal dysfunction [6–9], and a combined creatinine-cystatin C equation has been suggested for classification of the estimated glomerular filtration rate (eGFR) instead of creatinine alone [6]. A number of studies have demonstrated that cystatin C is closely associated with cardiovascular disease, including incident congestive heart failure [9] and carotid atherosclerosis [10], and is significantly associated with the presence and severity of asymptomatic coronary artery disease in metabolic syndrome patients with normal kidney function [11]. Moreover, the association between serum cystatin C and the risk of progression from normoglycaemia to prediabetes has been reported [12]. To date, the relationship between cystatin C and multivessel disease in diabetes mellitus patients remains unclear. Therefore, this study was conducted to explore the role of cystatin C in multivessel coronary artery disease in type 2 diabetes patients with normal renal function and to determine whether cystatin c influences multivessel coronary artery disease independent of traditional risk factors. In addition, we investigated whether cystatin C can serve as a biomarker to predict the presence of multivessel coronary artery disease.

## Methods

### Study subjects

A total of 135 type 2 diabetes patients and 179 age-matched subjects without diabetes with a normal estimated glomerular filtration rate (eGFR) were enrolled in this cross-sectional study. All patients were admitted to QiLu Hospital of Shandong University (Jinan, China) and were referred for elective coronary angiography for suspected coronary artery disease by cardiologists from March 2010 to March 2012. None of the patients in this study suffered from malignancy, inflammatory disease, valvular heart disease, renal dysfunction (creatinine-based eGFR < 90 mL/min/1.73 m^2^, serum creatinine> 133 μmol/L), or hepatic dysfunction. Large doses of glucocorticoids enhance cystatin C [13]. Thyroid dysfunction also influences cystatin C levels [14, 15]. Therefore, individuals with these factors were also excluded from this study. Written informed consent was obtained from all subjects. The study was performed in compliance with the guidelines for the Declaration of Helsinki and was approved by medical ethics regulations of the Medical Ethical Committee of Qilu Hospital of Shandong University (Jinan, China).

### Diagnostic criteria

(1) Type 2 diabetes mellitus was defined as fasting plasma glucose > 7 mmol/l, 2-h post-challenge plasma glucose > 11.1 mmol/l, or current use of anti-diabetic drugs, based on the diagnostic criteria set forth by the American Diabetes Association [16].

(2) Hypertension was defined as systolic blood pressure or diastolic blood pressure (SBP/DBP) ≥ 140/90 mmHg and/or diagnosed hypertension treated with antihypertensive therapy [17].

### Clinical and biochemical assessment

Cardiovascular risk factors and demographic characteristics were obtained from medical records. Cardiovascular risk was assessed in terms of smoking status (≥20 packs per year) and alcohol status. BMI was calculated as weight (kg) divided by the square of the height (m^2^). Systolic and diastolic blood pressures were measured using standard methods.

All patients had their blood drawn in the morning after an 8-h fast. Serum was used to analyse low-density lipoprotein cholesterol (LDL-c), total cholesterol (TC), high-density lipoprotein cholesterol (HDL-c), uric acid (UA), triglycerides (TGs) and fasting plasma glucose (FPG) using an automatic biochemistry analyser (Roche Cobas 8000 modular analyser Series C701, Mannheim, Germany). The concentration analysis of cystatin C was the same as described in a previous paper [11]. eGFR was estimated with the CKD-EPI equation [18]. Von Clauss methods were used to detect fibrinogen, and serum creatinine (sCr) levels were measured using an enzymatic method. Patients with an eGFR< 90 mL/min/1.73 m^2^ were excluded.

### Coronary angiography

A standard technique was used for coronary angiography. Two experts who were not familiar with the study performed the angiography. Coronary heart disease was diagnosed when angiography showed at least one lesion with lumen stenosis ≥50%. The severity of coronary atherosclerosis was based on the Gensini score [19]. Multiple multivessel disease was defined as > 50% luminal narrowing in the left main coronary artery or in more than three sites of coronary arteries.

### Statistical analysis

The Kolmogorov-Smirnov test and Levine test were used to evaluate the normality and variance uniformity of the data. The continuous variables with a normal distribution are expressed as the mean ± standard deviation, and the classification variables are expressed as percentages. Nonnormally distributed variables such as TG and Gensini scores are represented by the median and the quartile range. Chi-square tests were used to compare categorical variables. Normally distributed variables were tested by unpaired Student’s t test. Age, BMI, hypertension, sex, smoking status, blood lipids (TC, TGs, LDL-c, HDL-c) and eGFR were identified as potential confounding factors and were adjusted in the model. Spearman correlation analysis was used to determine the factors related to the Gensini score. Logistic regression analysis was used to assess the relationship between cystatin C and the presence of multivesicular disease. Biomarker cutoff points for multiple vessel disease were analysed by the receiver operating characteristic (ROC) curve. SPSS 16.0 was used to analyse the data (SPSS, Chicago, IL, USA). The significance level was set as a two-tailed *p* < 0.05.

## Results

### Characteristics of individuals with and without diabetes

**Fig. 1:**
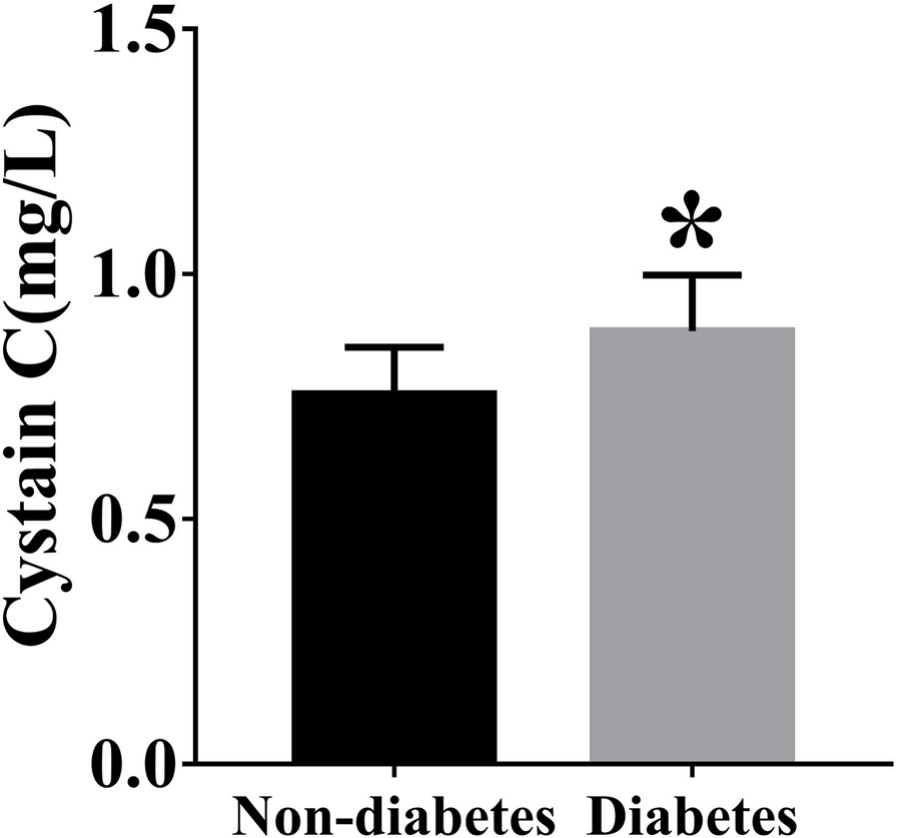
Serum cystatin C levels between CAD patients with diabetes (n = 135) and those without diabetes (n = 179). CAD, coronary artery disease. *P < 0.01 vs. Non-diabetes group

Among the 135 diabetes and 179 nondiabetic patients who participated in this study, the UA and FPG levels were significantly higher in patients with diabetes than in those without diabetes (*P* = 0.003, *P*<0.001, respectively). There was no difference in age, sex ratio, blood pressure, BMI or lipid profile between the two groups (Table 1). The rates of hypertension and smoking status in patients also showed no difference. Among the patients with CAD, those with diabetes mellitus had significantly higher serum cystatin C levels than those without diabetes mellitus (0.75 ± 0.10 vs 0.89 ± 0.11 mg/L, *P*<0.001; Fig. 1).

**Table 1:** Clinical and biochemical characteristics of the study subjects

	Non-diabetes (***n*** = 179)	Diabetes (***n*** = 135)	p
**Age (years)**	54.74 ± 8.759	56.41 ± 8.20	0.81
**Males% (n)**	72 (129)	65 (88)	0.15
**smoking%(n)**	48 (86)	48 (65)	0.90
**Hypertension%(n)**	63 (113)	72 (97)	0.25
**drinking %(n)**	48 (81)	50 (68)	0.66
**BMI (kg/m**^**2**^**)**	26.32 ± 4.42	26.99 ± 3.06	0.15
**Systolic BP (mmHg)**	132.39 ± 17.93	138.01 ± 22.69	0.08
**Diastolic BP (mmHg)**	78.55 ± 12.70	80.36 ± 13.39	0.20
**FPG (mmol/L)**	5.27 ± 1.27	8.39 ± 2.57	<0.001
**TC (mmol/L)**	4.81 ± 1.07	4.71 ± 0.88	0.42
**TG (mmol/L)**	1.91 ± 1.13	2.26 ± 1.41	0.09
**LDL-c (mmol/L)**	2.98 ± 0.88	2.82 ± 0.71	0.09
**HDL-c (mmol/L)**	1.14 ± 0.24	1.12 ± 0.88	0.851
**SCr (μmol/L)**	64.13 ± 9.97	62.27 ± 10.18	0.09
**eGFR (ml/min/1.73m**^**2**^**)**	103.11 ± 7.22	102.83 ± 7.50	0.72
**UA (μmol/L)**	314.82 ± 74.65	382.56 ± 72.67	0.003
**Fibrinogen(g/L)**	3.48 ± 1.12	3.58 ± 0.81	0.41
**Cystatin C (mg/L)**	0.75 ± 0.10	0.89 ± 0.11	<0.001
**Gensini score**	16.5 ± 20.04	41.25 ± 33.38	<0.05

*BMI* body mass index; *BP* blood pressure; *FPG* fasting plasma glucose; *TC* total cholesterol; *TG* triglyceride; *LDL-c* low-density lipoprotein cholesterol; *HDL-c* high-density lipoprotein cholesterol; *SCr* serum creatinine; *UA* uric acid. Data are means ± SD or median (interquartile range)

### Correlation between serum cystatin C and CAD severity

**Fig. 2:**
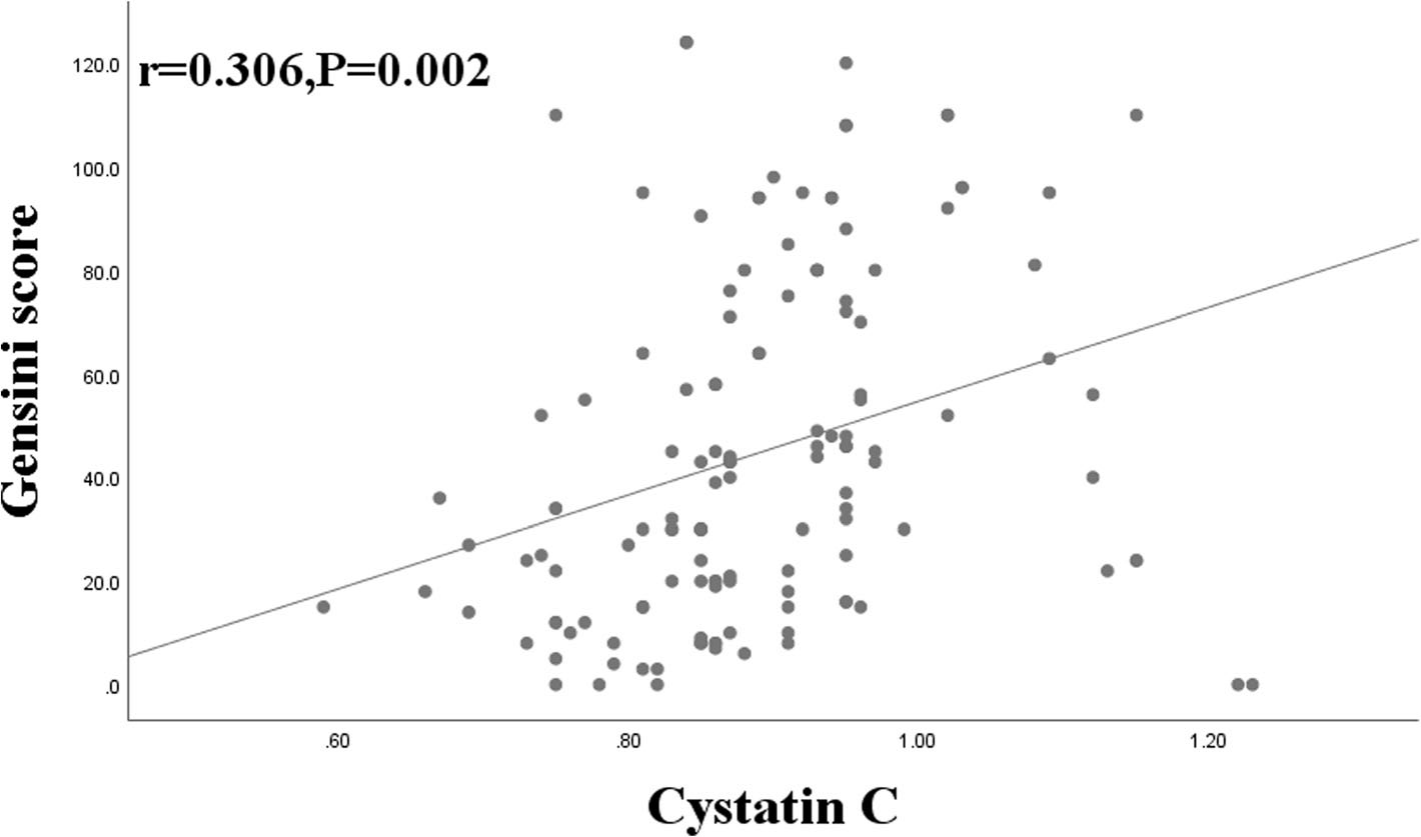
Correlation between serum cystatin C and the Gensini score, in diabetes mellitus patients with normal renal function. CAD, coronary artery disease

The relationship between serum cystatin C levels and Gensini score, which represents the extent of multivessel disease, was investigated in the diabetes group (Fig. 2). Spearman correlation analysis showed that serum cystatin C, BMI, fibrinogen and fasting plasma glucose (FPG) were significantly correlated with the Gensini score (r = 0.306, r = 0.217, r = 0.246, r = 0.282, *P* = 0.002*, P* = 0.032 *P* = 0.017, *P* = 0.004, respectively), there was no relationship between HDL-c and the Gensini score (*P* = 0.162) (Table 2).

**Table 2:** Correlation of Gensini score with clinical and biological parameters

Variables	Gensini score	
	r	p
**Cystatin C (mg/L)**	0.306	0.002
**BMI (kg/m**^**2**^**)**	0.217	0.032
**Fibrinogen(g/L)**	0.246	0.017
**FPG (mmol/L)**	0.282	0.004
**HDL-c (mmol/L)**	0.055	0.162

*BMI* body mass index; *BP* blood pressure; *FPG* fasting plasma glucose; *HDL-c* high-density lipoprotein cholesterol;

### Diagnostic power of cystatin C for multivessel disease identified by coronary angiography in type 2 diabetes mellitus patients

Multiple logistic regression analysis was applied to assess the usefulness of cystatin C as a diagnostic marker for the presence of multivessel disease after adjusting for conventional CAD risk factors, including age, sex, BMI, smoking status, hypertension, TC, LDL-c, HDL-c, TGs, SBP, DBP, fibrinogen, SCr, FPG, eGFR and cystatin C. Before Multiple logistic regression analysis, we performed collinearity diagnosis, and the VIF value of all our variables was less than 3.0. Serum cystatin C (odds ratio, OR = 2.21, 95% confidence interval (CI): 1.316–3.727, *P* = 0.003) and FPG (OR = 1.28, 95% CI: 1.045–1.565, *P* = 0.017) were found to be independent predictors of the presence of multivessel disease (Table 3).

**Table 3:** Multiple logistic regression analysis indicating factors independently associated with multi-vessel disease in type 2 diabetes mellitus

Parameters	B	SE	OR	95% CI	p
**FPG**	0.246	0.103	1.28	1.045–1.565	0.017
**Cystatin C**	0.795	0.266	2.21	1.316–3.727	0.003

B regression coefficient; *SE* standard error; *CI* confidence interval; *OR* odds ratio; *FPG* fasting plasma glucose. Variables included in the model are age, sex, BMI, smoking, alcohol hypertension, and biochemical risk factors TC, LDL-c, HDL-c, TG, SBP, DBP, fibrinogen, SCr, FPG, eGFR and cystatin C

**Fig. 3:**
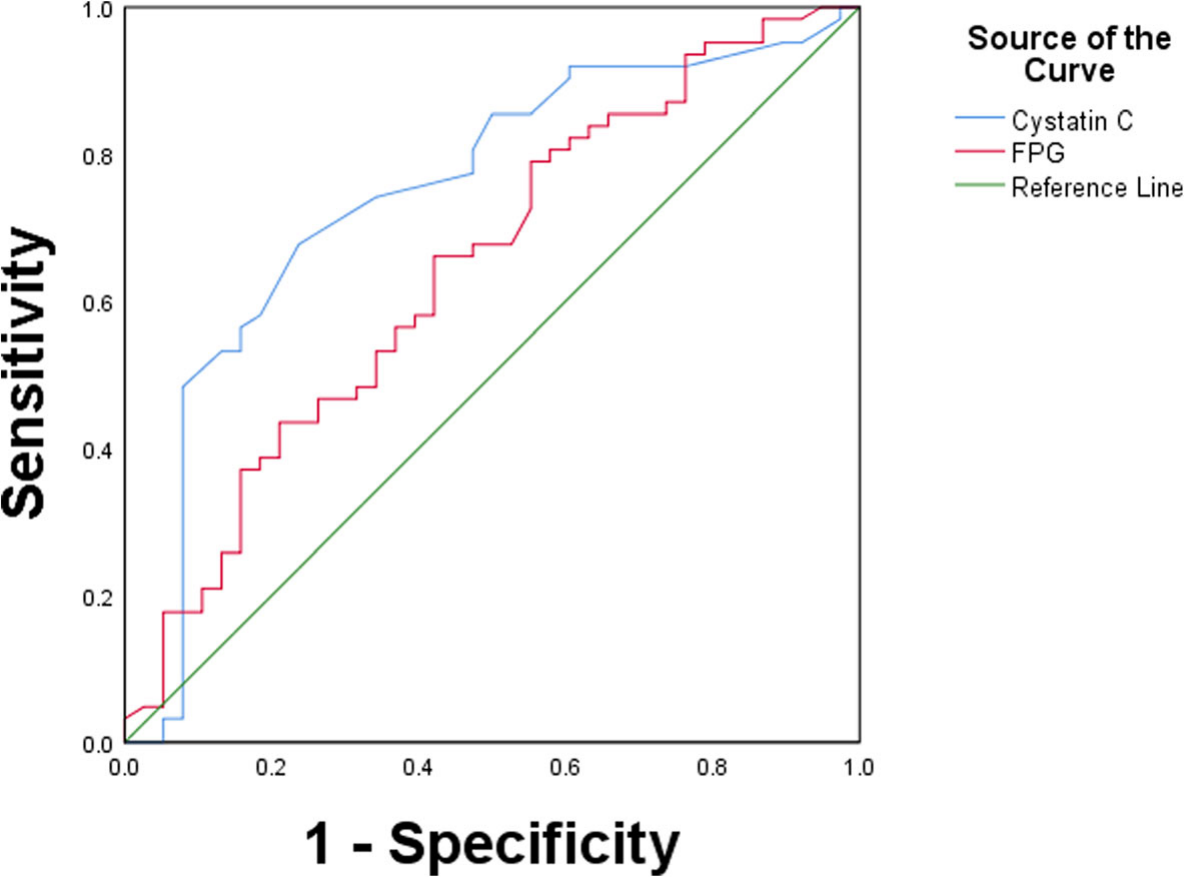
Receiver operating characteristic curve to determine the cutoff value of cystatin C and FPG for multi-vessel disease in diabetes mellitus patients with normal renal function. FPG: fasting plasma glucose

The optimal cut-off value for the diagnosis of multivessel disease was determined according to the ROC curves. A serum cystatin C level of ≥0.865 mg/L predicted the presence of multivessel disease with a sensitivity of 67.7%, specificity of 76.3% and an area under the curve of 0.748 (95% CI: 0.645–0.851, *P* < 0.001, Fig. 3) (Table 4).

**Table 4:** Cut-off value, sensitivity, specificity, and area under the curves for biomarkers in predicting for multi-vessel disease in type 2 diabetes mellitus

Biomarkers	Cut-off value	Sensitivity(%)	Specificity(%)	AUC	95% CI	P
**FPG (mmol/L)**	6.740	79.0	44.7	0.644	0.531–0.756	0.016
**Cystatin C (mg/L)**	0.865	67.7	76.3	0.748	0.645–0.851	0.000

*FPG* fasting plasma glucose; *CI* confidence interval; *AUC* area under curve

## Discussion

In this cross-sectional study, we found a positive relationship between the serum levels of cystatin C and multivessel disease by coronary angiography in type 2 diabetes mellitus patients with normal renal function. Serum cystatin C levels were significantly positively correlated with the Gensini score. In addition, higher serum cystatin C could independently predict the presence of multivessel disease by coronary angiography. The ability of cystatin C to distinguish multivessel disease patients among patients with type 2 diabetes mellitus and normal renal function was moderate. Therefore, cystatin C was a useful biomarker for the identification of multivessel disease in subjects with diabetes mellitus. To the best of our knowledge, this is the first study to demonstrate the usefulness of cystatin C as a diagnostic tool for multivessel disease and to suggest an optimal cutoff value in diabetes patients with normal renal function.

Serum cystatin C has been shown to be a new risk factor for CAD. Numerous prior studies have suggested that patients with elevated cystatin C have greater cardiovascular risk and a greater risk of mortality [20–23]. Cystatin C has been proposed to be not only a sensitive marker of kidney dysfunction but also a biomarker of atherosclerosis and cardiovascular risk. Animal studies have revealed that cystatin C might interact with the inflammatory response, leading to activation of cathepsins and resulting in the degradation of collagen in the atheroma plaque, leading to an increased risk of rupture [22].

Our results indicate that diabetes patients had higher serum cystatin C levels than nondiabetic patients. This may be explained, at least partly, by the close association of cystatin C with insulin resistance, obesity and hypertension conditions, which are closely related to diabetes; these associations have been shown in several studies [24–26]. This result is very similar to the report by Servais et al. [27], which included 925 dyslipidaemic subjects, including 11% diabetic patients. HOMA-IR increased in proportion to cystatin C quartiles and showed an independent association with cystatin C by multiple regression analysis. Because insulin resistance is an important element in the development of type 2 diabetes mellitus, these findings suggest that insulin resistance may play an additional role in the link between cystatin C and type 2 diabetes mellitus, although the underlying mechanism remains unclear. Recently, K. Sahakyan et al. [28] found an association of serum cystatin C with the incidence of type 2 diabetes mellitus in a long-term population-based study, which further confirmed the close relationship of cystatin C with diabetes mellitus.

We also found a positive association between serum cystatin C levels and the Gensini score independent of eGFR, even after adjustment for established risk factors associated with cardiovascular disease and cystatin C. Niccoli et al. [29] demonstrated that the independent association between cystatin C and CAD severity was superior to that of creatinine or eGFR, and we obtained similar results. Likewise, Mevlut Koc et al. [30] showed a strong correlation between cystatin C and the severity of CAD. In this cross-sectional study, cystatin C was suggested to be a useful laboratory tool in predicting the presence and severity of CAD in daily practice.

Vascular disease that affects macrovasculature is an important cause of morbidity and mortality in diabetic patients. A simple and noninvasive tool for predicting multivessel disease in type 2 diabetes patients is needed. Many studies have been conducted to assess the usefulness of cystatin C in predicting cardiovascular risks and renal dysfunction [31–33], and various cutoff values have been suggested in multiple clinical settings. However, the optimal cutoff value in detecting multivessel disease in patients with type 2 diabetes mellitus and normal renal function has not been suggested to date. We analysed the optimal cut-off value of cystatin C in our study, which indicated an optimal cut-off of 0.865 mg/L with 67.7 and 76.3% sensitivity and specificity, respectively. Our results indicated that cystatin C is a stronger predictor of multivessel disease than fasting plasma glucose. Serum cystatin C might be a powerful diagnostic tool to screen for multivessel disease in patients with diabetes mellitus and normal renal function.

Several limitations should be considered when our results are interpreted. First, the sample size of our study was relatively small, and we did not consider medication in our analyses. Second, we did not collect 24-h urine for direct measurements of GFR because of its difficulty in daily practice. However, numerous studies have demonstrated that the creatinine-based eGFR provides a good approximation. Third, given its cross-sectional design, a causal relationship between cystatin C indices and multivessel disease prevalence cannot be inferred.

## Conclusion

In summary, our findings demonstrate that cystatin C was significantly associated with the severity of CAD and independently predicted the presence of multivessel disease in patients with diabetes mellitus with normal renal function. Cystatin C emerged as a potential marker for identifying multivessel disease in populations that may benefit from therapeutic strategies aimed at preventing the development of multivessel coronary artery disease. Further research is warranted to clarify the pathophysiologic mechanisms responsible for these associations.

## Data Availability

All data generated or analysed during this study are included in this published article and its additional information fles.

## Abbreviations

CAD: Cardiovascular diseases
eGFR: Estimating glomerular filtration rate
DM: Diabetes mellitus
SBP: Systolic blood pressure
DBP: Diastolic blood pressure
BMI: Body mass index
HOMA-IR: Homeostasis model assessment of insulin resistance
